# 1,5-Anhydroglucitol, an indicator of short term glycaemic control, is the most discriminatory metabolomic marker in adolescents with type 1 diabetes compared to control subjects

**DOI:** 10.1186/1687-9856-2015-S1-P2

**Published:** 2015-04-28

**Authors:** Louise S Conwell, Mark P Hodson, Panagiotis K Chrysanthopoulos, Ristan M Greer, Lars K Nielsen, Tracey Baskerville

**Affiliations:** 1 Department of Endocrinology and Diabetes, Royal Children’s Hospital, Brisbane, QLD, Australia; 2 Queensland Children’s Medical Research Institute, University of Queensland, Brisbane, QLD, Australia; 3 School of Medicine, University of Queensland, Brisbane, QLD, Australia; 4 Metabolomics Australia, Australian Institute for Bioengineering and Nanotechnology, Brisbane, QLD, Australia; 5 Department of Endocrinology and Diabetes, Mater Children’s Hospital, Brisbane, QLD, Australia

## Aim

To compare the metabolomic profile of adolescents with T1D (≥5 years duration) to controls using gas chromatography–mass spectrometry (GC-MS).

## Methods

Design Case control study. Setting Tertiary paediatric hospital clinic. Population 27 (14F/13M) adolescents with T1D (age (median, interquartile range) 15.5, 14.7-16.4 years; duration 7.7; 6.0-11.8 years; HbA1c 9.1, 8.1-10.1%); glucose 13.35 (7.60-17.85) and 27 (14F/13M) control participants (age 15.1, 14.4-16.8 years). BMI was <95^th^ percentile. Measures Fasting plasma and urine metabolomes were profiled by GC-MS and compared between cohorts. Statistics Univariate comparisons:-Spearman correlations, t-tests/Wilcoxon rank sum tests. Multivariate comparisons:-PCA, OPLS-DA and OPLS.

## Results

For GC-MS (plasma and urine), the molecule most influential in separating the two groups was identified as 1,5-anhydroglucitol (1,5-AG) a metabolically inert polyol that is a short-term marker of glycaemic control (7-14 days). It competes with glucose for reabsorption in the kidneys. Otherwise stable levels of 1,5-AG are rapidly depleted as blood glucose levels exceed the renal threshold for glycosuria. 1,5-AG was more influential on group classification than fasting glucose or HbA1c.

Multivariate regression modelling of the plasma data (glucose signals removed) was performed against glucose and HbA1c groups (glucose:4.2-5.4mmol/L, 5.3-9.9mol/L, 11.0-36.7mmol/L; HbA1c:4.7-6.0%, 7.3-9.2%, 9.9-15.4%). Three distinct groups emerged for each variable indicating clear metabolomic differences.

## Conclusion

Metabolomic profiling was feasible in this context. GC-MS revealed a “marker” distinguishing the two groups without any bias for or targeting of the analyte. The metabolic profile of the adolescents with diabetes appears to be most influenced by short-term (7-14days) hyperglycaemia. The planned GC-MS fatty acid methyl ester (FAME) analysis and Liquid Chromatography-MS will reduce inherent interference by glucose and provide a more comprehensive coverage of the metabolome.

A 1,5-AG blood assay (GlycoMark) is not yet routinely available in pathology laboratories in Australia. Due to discussions following this study, it may shortly become available. Its particular utility is to assess recent glycaemic control and suggest unrecognised postprandial hyperglycaemia in moderately-controlled (HbA1c 6.5-8%) patients.

